# Interleukin 4 induces apoptosis of acute myeloid leukemia cells in a Stat6-dependent manner

**DOI:** 10.1038/leu.2017.261

**Published:** 2017-09-22

**Authors:** P Peña-Martínez, M Eriksson, R Ramakrishnan, M Chapellier, C Högberg, C Orsmark-Pietras, J Richter, A Andersson, T Fioretos, M Järås

**Affiliations:** 1 Department of Clinical Genetics, Lund University, Lund, Sweden; 2 Department of Molecular Medicine and Gene Therapy, Lund University, Lund, Sweden; 3 Department of Hematology, Oncology and Radiation Physics, Skåne University Hospital, Lund, Sweden

## Abstract

Cytokines provide signals that regulate immature normal and acute myeloid leukemia (AML) cells in the bone marrow microenvironment. We here identify interleukin 4 (IL4) as a selective inhibitor of AML cell growth and survival in a cytokine screen using fluorescently labeled AML cells. RNA-sequencing of the AML cells revealed an IL4-induced upregulation of Stat6 target genes and enrichment of apoptosis-related gene expression signatures. Consistent with these findings, we found that IL4 stimulation of AML cells induced Stat6 phosphorylation and that disruption of *Stat6* using CRISPR/Cas9-genetic engineering rendered cells partially resistant to IL4-induced apoptosis. To evaluate whether IL4 inhibits AML cells *in vivo*, we expressed IL4 ectopically in AML cells transplanted into mice and also injected IL4 into leukemic mice; both strategies resulted in the suppression of the leukemia cell burden and increased survival. Notably, IL4 exposure caused reduced growth and survival of primary AML CD34^+^CD38^−^ patient cells from several genetic subtypes of AML, whereas normal stem and progenitor cells were less affected. The IL4-induced apoptosis of AML cells was linked to Caspase-3 activation. Our results demonstrate that IL4 selectively induces apoptosis of AML cells in a Stat6-dependent manner—findings that may translate into new therapeutic opportunities in AML.

## Introduction

Acute myeloid leukemia (AML) is a fatal disorder characterized by an accumulation of immature myeloid blasts in the bone marrow. Current treatment protocols have improved only modestly for the past 30 years and AML is associated with a poor overall survival, especially among the elderly (5-year overall survival of ~20% in patients 60 or more years old).^1, 2^

With increased understanding of how immature normal and leukemic cells interact with the microenvironment, new possibilities for intervening selectively against malignant cells are emerging.^3^ Successful examples in preclinical AML models comprise targeting of the leukemia microenvironment by CXCR4 inhibition to overcome resistance to kinase inhibitors and chemotherapy,^4^ and blocking of CD47 on AML cells to induce preferential phagocytosis of leukemia cells.^5^ In addition to cell–cell interactions, cytokines such as interleukin 6 (IL6) and tumor necrosis factor-α have been shown to positively regulate leukemia stem cells (LSCs),^6, 7^ a self-renewing cell population capable of initiating and propagating leukemia.^8^ Consistent with IL1 being a positive regulator of LSCs, we and others recently showed that blocking IL1 signaling inhibits LSCs.^9, 10, 11^ However, little is known about cytokines that negatively affect LSC growth and survival, and whether such cytokines can be utilized for novel treatment approaches in AML.

To identify endogenous suppressors of immature AML cells, we performed a cytokine screen and identified IL4 as a negative regulator of primitive AML cells. IL4 induced apoptosis of AML cells in a Stat6-dependent manner, thus revealing a previously unrecognized role of IL4 as an inhibitor of the growth and survival of primitive AML cells.

## Materials and methods

### Murine leukemia model

Murine *MLL-AF9* leukemia cells were propagated and harvested essentially as described before.^12, 13^ For details on the *in vivo* model and culture conditions, the colony-forming assay and the bone marrow-homing assay, see Supplementary Information.

### Cytokine screen

c-Kit^+^ dsRed^+^ leukemia cells and c-Kit^+^ murine normal bone marrow (NBM) cells were mixed and seeded into 96-well plates. The cells were cultured in a serum-free expansion medium (Stemspan, StemCell Technologies, Vancouver, BC, Canada) containing 1% penicillin/streptomycin supplemented with one cytokine (100 ng/ml) condition per well (114 murine cytokines; purchased from Prospec, East Brunswick, NJ, USA; Peprotech, Rocky Hill, NJ, USA; and R&D Systems, Minneapolis, MN, USA; listed in Supplementary Table 1). The cells were incubated for 72 h and cell numbers were determined by flow cytometry using CountBright beads (Life Technologies, Carlsbad, CA, USA).

### Flow cytometric analysis and cell sorting

The flow cytometric analyses were performed using a FACS Canto II (BD Biosciences, San Jose, CA, USA) or a FACS LSRFortessa (BD Biosciences), and cell sorting was performed using a FACS Aria II (BD Biosciences). For details on the cell cycle analysis, apoptosis analysis and antibodies used, see Supplementary Information.

### Competitive bone marrow transplantations

C57BL/6, B6SJL (both from Taconic, Hudson, NY, USA) and C57BL/6 × B6SJL (in-house breeding) were used for the experiments. For details on the experimental design, see Supplementary Information.

### Viral vectors

For details on experiments involving the murine stem cell virus gammaretroviral vector coexpressing a m*IL4* cDNA, and lentiviral vectors expressing Cas9^+^ or a *Stat6* single-guide RNA (sgRNA), see Supplementary Information.

### RNA-sequencing analysis

Global gene expression profiling was performed on c-Kit^+^ leukemia cells and c-Kit^+^ NBM cells cultured for 18 h, as well as freshly isolated c-Kit^+^ leukemia cells. For details on the RNA extraction, library preparation and analysis, see Supplementary Information. Raw data and normalized gene expression data are available in the Gene Expression Omnibus database under accession number GSE79068.

### Human AML and NBM samples

Mononuclear cells from AML patients and healthy volunteers were obtained after informed consent using ethical permits approved by the Regional Ethics Committee at Lund University. Mononuclear cells were separated using Lymphoprep (Axis Shield PoC AS, Dundee, UK), and CD34^+^ cells were enriched using the human CD34 MicroBead Kit (Miltenyi Biotec, Bergisch Gladbach, Germany). For culture conditions, see Supplementary Information. Patient data are summarized in Supplementary Table 2.

### Statistical analyses

Prism 6 (Graphpad Software, La Jolla, CA, USA) was used for statistical analyses including Student’s *t*-tests, analyses of variance, linear regressions and Kaplan–Meier survival analysis. Significance is depicted with asterisks: *P<0.05, **P<0.01, ***P<0.001, ****P<0.0001. Data are presented as mean±s.d.'s.

## Results

### Cytokine screening identifies IL4 as an inhibitor of primitive AML cells

**Figure 1:**
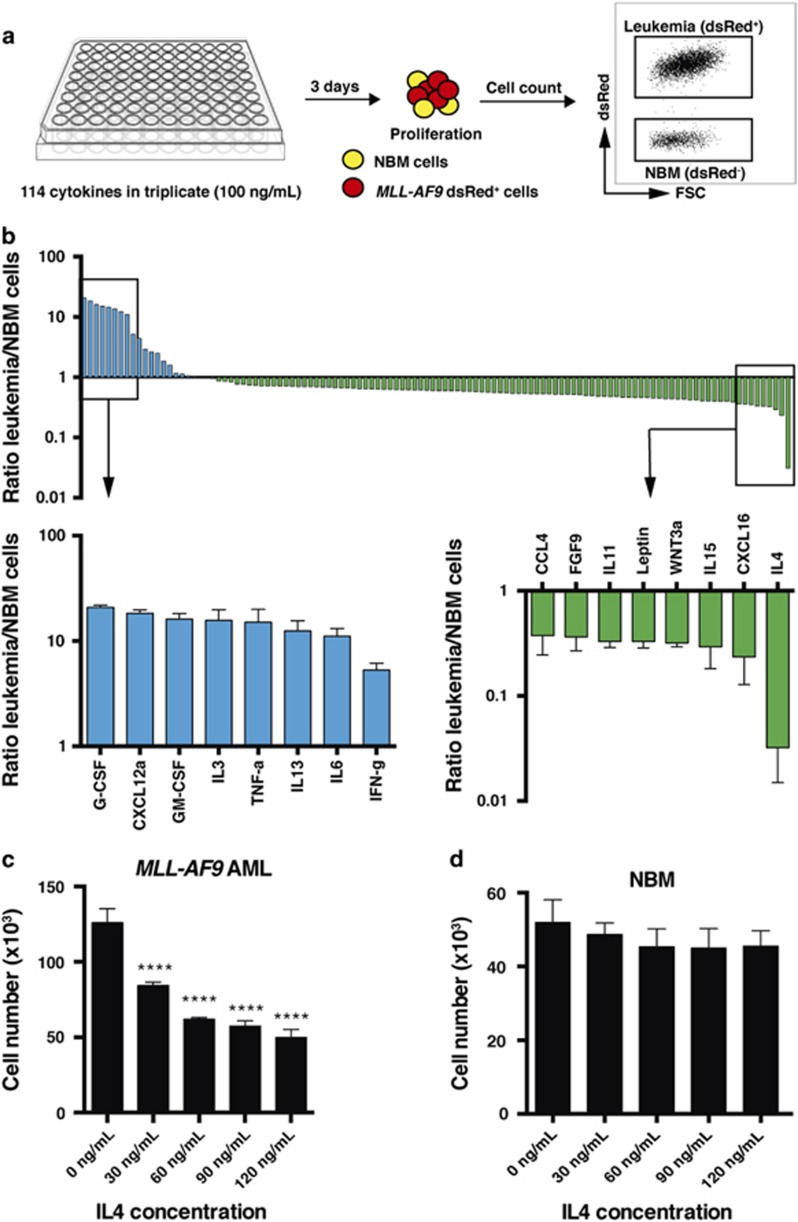
Cytokine screening identifies IL4 as a negative regulator of *MLL-AF9* leukemia cells. (**a**) Schematic diagram showing the arrayed cytokine screen with 10 000 seeded c-Kit^+^ dsRed^+^ AML cells mixed with 10 000 seeded c-Kit^+^ NBM cells per well, performed in 96-well plates, and without any baseline cytokines added to the medium. A cytokine library of 114 recombinant murine proteins was used, each tested separately. Cell number was determined using flow cytometry after 3 days of culture (*n*=3). (**b**) Ranked screening results presented as the ratio in output cell number between leukemia (dsRed^+^) and NBM cells for each cytokine screened. Data from the full screen (blue, selective positive regulators of AML cells versus NBM cells; green, selective negative regulators of AML cells versus NBM cells). (**c**) Output cell number of 10 000 seeded c-Kit^+^ *MLL-AF9* leukemia cells, and (**d**) 10 000 c-Kit^+^ NBM cells, following dose titration with mIL4 for 3 days with mIL3 (20 ng/ml) as a baseline condition (*n*=3). NBM, normal bone marrow. ****P<0.0001.

To identify cytokines acting as selective negative regulators of primitive AML cells affecting their growth and survival, we performed an *in vitro* cytokine screen using dsRed^+^ c-Kit^+^ murine AML cells mixed with c-Kit^+^ NBM cells, allowing for fluorescence-based separation of the two cell types (Figure 1a). The dsRed^+^ leukemia cells express the *MLL-AF9* fusion gene and we have successfully used these cells previously in screens as they have a well-defined LSC population and initiate AML with short latency, enabling rapid follow-up experiments in syngeneic hosts.^12, 13, 14, 15^

Primitive NBM cells and *MLL-AF9* leukemic cells were enriched by c-Kit selection, whereafter we assessed the effect of 114 murine cytokines in mixed cultures (Figure 1a and Supplementary Table 1). We used a cytokine concentration of 100 ng/ml, as it has previously been used in similar screens.^16^ As a proof of principle, the screen identified several cytokines as selective positive regulators of leukemia cells previously associated with leukemic stem and progenitor cell biology, including CXCL12a (SDF1), IL3, tumor necrosis factor α and IL6 (Figure 1b).^6, 7, 17, 18^ In addition, several selective negative regulators of AML cells were identified and, of these, IL4 elicited the strongest depletion of leukemia cells (Figure 1b). Validation experiments demonstrated that the negative effect of IL4 on leukemia cell expansion was observed even in the presence of cytokines such as IL3, which gives strong proliferative signals, and without NBM cells present (Figure 1c). Moreover, IL4 inhibited colony-forming leukemia cells (Supplementary Figure 1). By contrast, IL4 did not affect the growth and survival of primitive NBM cells (Figure 1d).

### IL4 induces apoptosis of AML cells in a p53-independent manner

**Figure 2:**
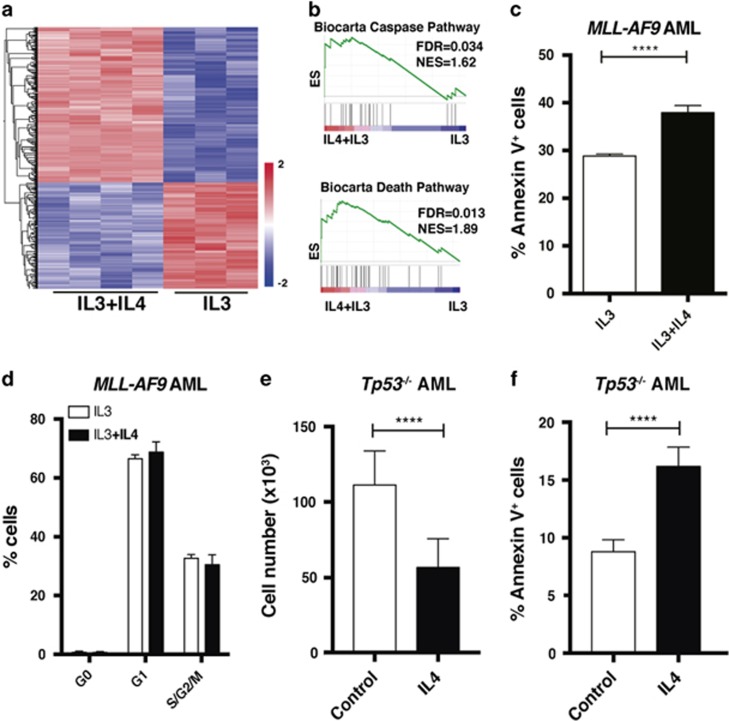
RNA sequencing reveals an IL4 signature in *MLL-AF9* leukemia cells enriched for apoptosis-related gene expression. c-Kit^+^ *MLL-AF9* AML cells were cultured with mIL4 and mIL3 or mIL3 only (control). (**a**) RNA sequencing of murine AML cells stimulated for 18 h with mIL4. A two-group comparison between control and IL4-treated AML cells revealed an IL4-induced signature of 390 differentially expressed genes (FDR<0.05, *P*<0.001). Genes in the heatmap are ordered by hierarchical clustering. (**b**) Gene set enrichment analysis (GSEA) showing that the IL4 gene expression signature in AML cells is enriched for Caspase and death pathways (Biocarta). (**c**) Apoptosis and (**d**) cell cycle analysis of leukemia cells treated with mIL4 for 3 days (*n*=3). (**e**) Output cell number of 10 000 seeded c-Kit^+^ *Tp53*^−*/*−^ *MLL-AF9* leukemia cells treated with mIL4 or control for 3 days (*n*=3). (**f**) Apoptosis analysis of c-Kit^+^ *Tp53*^−*/*−^ *MLL-AF9* leukemia cells treated with mIL4 or control for 3 days (*n*=3). ES, enrichment score; FDR, false discovery rate; NES, normalized enrichment score. ****P<0.0001.

To explore the biological mechanism whereby IL4 exerts its negative effect on leukemia cells, we stimulated both c-Kit^+^ *MLL-AF9* AML and c-Kit^+^ NBM cells with IL4 and performed RNA sequencing. IL4 induced distinct gene expression signatures in the two cell populations (Figure 2a and Supplementary Figure 2a and b). To link the IL4-induced gene expression patterns in AML cells to previously reported gene expression signatures, we carried out gene set enrichment analysis.^19^ We found that the IL4 signature in leukemia cells was enriched for genes involved in cell death and Caspase pathway signatures (Figure 2b and Supplementary Tables 3 and 4). Consistent with these findings, IL4 stimulation forced the AML cells into apoptosis as evident by an increase in Annexin V^+^ cells (Figure 2c and Supplementary Figures 3a and b), whereas their cell cycle or differentiation status was not affected (Figure 2d and Supplementary Figures 3c and d). Interestingly, by sorting the top 10% and lowest 10% IL4ra-expressing cells and exposing them to IL4, we found that a higher IL4ra expression level sensitized the AML cells for the antileukemic effects of IL4 (Supplementary Figures 4a–c). By contrast, IL4 stimulation of NBM cells did not induce a gene expression signature that was enriched in any of the gene sets that matched with the IL4 signature in AML cells (false discovery rate<0.05; Supplementary Table 3). Moreover, we assessed which components of the IL4R complexes that are expressed in c-Kit^+^ leukemia cells, and found that the *Il4ra* and *Il2rg* are expressed, but not *Il13ra1* (Supplementary Figure 5).

To address whether IL4-induced apoptosis is mediated by p53, a well-known regulator of apoptosis,^20^ we used murine *Tp53*^−*/*−^ *MLL-AF9* leukemia cells, previously generated by us.^13^ We found that similar to *Tp53*^*+/+*^ AML cells (Figure 2c), IL4 forced *Tp53*^−*/*−^ AML cells into apoptosis resulting in reduced cell numbers upon culture (Figures 2e and f). These findings demonstrate that IL4 induces apoptosis of AML cells in a p53-independent manner.

### IL4 induces apoptosis of AML cells in a Stat6-dependent manner

**Figure 3:**
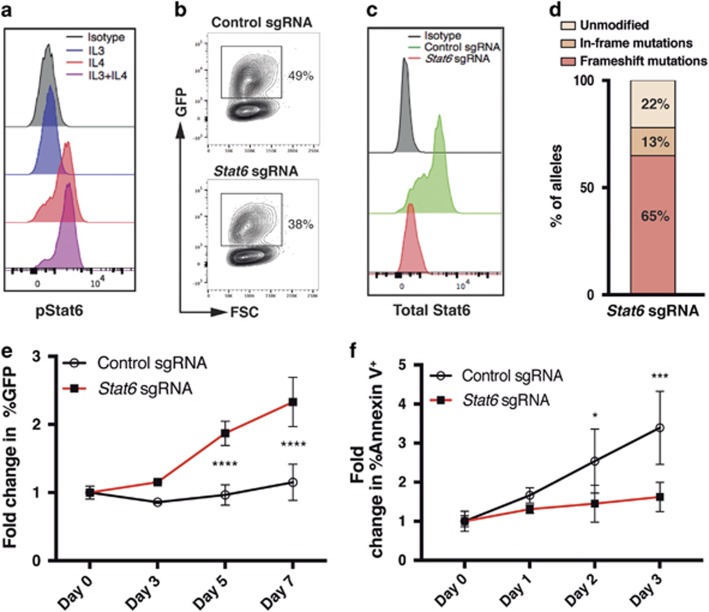
IL4 induces apoptosis of AML cells in a Stat6-dependent manner. (**a**) Staining of phosphorylated Stat6 (pStat6) upon stimulation with indicated cytokines in *MLL-AF9* leukemia cells using flow cytometry. (**b**) Green fluorescent protein (GFP) expression in Cas9^+^ *MLL-AF9* leukemia cells following transduction with lentiviral vectors coexpressing *Stat6* or control sgRNAs and GFP. (**c**) Stat6 expression within GFP^+^ cells 7 days post transduction. (**d**) Genetic alterations in *Stat6* detected by next-generation sequencing within sorted GFP^+^ cells 3 days post transduction. (**e**) Fold change in the percentages of GFP^+^ (sgRNA^+^) cells within each group during mIL4 treatment relative to day 0 (*n*=3). (**f**) Fold change in the percentages of Annexin V^+^ cells within each group during IL4 treatment relative to day 0 (*n*=3). *P<0.05, ***P<0.001, ****P<0.0001.

To further explore the molecular mechanism underlying the antileukemic effect of IL4, we measured phosphorylation of Stat6, one of the downstream effectors of the IL4 receptor,^21^ upon IL4 treatment. We found that short exposure of leukemia cells to IL4 resulted in phosphorylation of Stat6 (Figure 3a). Moreover, Stat6-target genes were enriched in the IL4 signature of AML cells as determined by gene set enrichment analysis (GSEA; Supplementary Table 3). To investigate whether the IL4-induced Stat6 activation was responsible for the depletion of leukemia cells, we first introduced ectopic Cas9 expression in the leukemia cells and sequentially a *Stat6* sgRNA (Figure 3b). The sgRNA directed to *Stat6* effectively suppressed Stat6 expression (Figures 3c and d). To assess whether *Stat6* disruption rendered the leukemia cells resistant to IL4, we monitored the percentage of sgRNA-expressing cells by tracking GFP^+^ cells during IL4 stimulation. The leukemia cells that expressed the *Stat6* sgRNA had a selective proliferative advantage upon IL4 treatment and exhibited reduced apoptosis, demonstrating that IL4 depletes the leukemia cells in a Stat6-dependent manner by inducing apoptosis (Figures 3e and f).

### IL4 selectively inhibits LSCs versus normal hematopoietic stem cells

**Figure 4:**
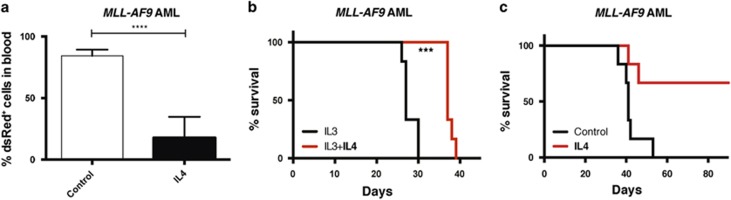
IL4 inhibits leukemia-initiating cells. (**a**, **b**) Overall, 500 000 c-Kit^+^ *MLL-AF9* AML cells were cultured *ex vivo* with mIL4 and mIL3 or mIL3 only (control) for 3 days, and then all treated cells were transplanted into sublethally irradiated mice (six mice per group). (**a**) Percentage of leukemic (dsRed^+^) cells in peripheral blood 14 days after transplantation. (**b**) Kaplan–Meier curves showing the survival of the mice. (**c**) In all, 10 000 c-Kit^+^ *MLL-AF9* AML cells were cultured *ex vivo* for 3 days with mIL4 or no cytokines (control), and then on average 500 viable cells per mouse were transplanted into sublethally irradiated mice for both groups (six mice per group). The Kaplan–Meier curves show the survival of the mice. ***P<0.001, ****P<0.0001.

Following the identification of IL4 as a negative regulator of c-Kit^+^ AML cells *in vitro*, we next evaluated whether IL4 is also capable of inhibiting the growth and survival of leukemia-initiating cells. c-Kit^+^ *MLL-AF9* leukemia cells were cultured *ex vivo* for 3 days with or without IL4, and then transplanted into sublethally irradiated recipient mice. Notably, at 2 weeks after transplantation, we observed more than fourfold lower levels of leukemic cells in the peripheral blood of mice receiving IL4-treated cells, hereafter referred to as the IL4 group (Figure 4a). The IL4 groups survived longer than the control groups (Figures 4b and c and Supplementary Figure 6), suggesting that IL4 inhibited leukemia-initiating cells. Moreover, we found that IL4 suppressed homing of the leukemia cells to the bone marrow as assessed 24 h post transplantation (Supplementary Figure 7). To evaluate whether IL4 affects normal hematopoietic stem and progenitor cell (HSPC) function, we next cultured LSK bone marrow cells with or without IL4 and performed competitive bone marrow repopulation experiments (Supplementary Figure 8a). In contrast to the negative effect of IL4 on LSCs, IL4 treatment of normal HSPC cells did not significantly alter their short- or long-term bone marrow repopulating capacity, or affect their lineage fate (Supplementary Figures 8b–e).

### IL4 is a negative regulator of AML cells *in vivo*

**Figure 5:**
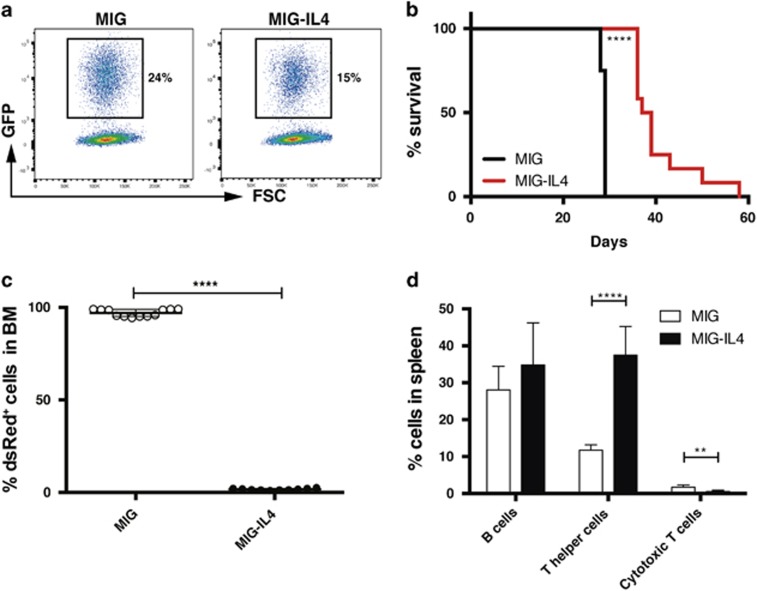
Ectopic expression of IL4 in AML cells *in vivo* has antileukemic effect. c-Kit^+^ *MLL-AF9* AML cells were transduced with retroviral vectors coexpressing a mIL4 cDNA (MIG–IL4) or a control vector (MIG) and GFP. Two days later, 30 000 sorted GFP^+^ cells were transplanted into sublethally irradiated mice. (**a**) GFP expression 2 days post transduction with the MIG and MIG–IL4 vectors. (**b**) Kaplan–Meier survival curves (12 mice per group). (**c**) Percentage of leukemia (dsRed^+^) cells in the bone marrow (BM) of mice at the time of killing. (**d**) Percentage of B cells (B220^+^), T helper cells (CD3^+^CD4^+^) and cytotoxic T cells (CD3^+^CD8^+^) within dsRed^−^ (non-leukemic) cells in spleens of mice transplanted with IL4-secreting leukemia cells or controls (six mice per group). **P<0.01, ****P<0.0001.

To address whether IL4 inhibits AML cells also *in vivo*, we generated a retroviral vector overexpressing mIL4 together with GFP (Figure 5a). Transductions of AML cells resulted in secretion of IL4 in the culture medium and about a 12-fold reduction in cell numbers upon 10-day cultures (Supplementary Figures 9a and b). Next, we tested whether constitutive IL4 expression had a negative effect on AML cells also *in vivo* by transplanting sorted GFP^+^ leukemia cells secreting IL4 shortly after transduction. At 2 weeks after transplantation, mice injected with IL4-secreting leukemia cells had elevated IL4 serum levels and reduced levels of leukemia cells in peripheral blood (Supplementary Figures 9c and d). The IL4 group survived significantly longer than the controls (median 38 versus 29 days, *P*<0.0001; Figure 5b). At the time of killing, mice in the IL4 group were almost devoid of leukemia cells in the bone marrow (mean 1.8% in the IL4 group versus 97% in the control group, *P*<0.0001; Figure 5c) and spleen (mean 3.8% in the IL4 group versus 76% in the control group, *P*<0.0001; Supplementary Figure 9e), and had elevated levels of IL4 in the bone marrow (Supplementary Figure 9f), demonstrating that IL4 had strong antileukemic effects on AML cells in an *in vivo* context. Moreover, mice in the IL4 group had splenomegaly with an increase in CD4^+^ T cells, consistent with a role of IL4 in promoting T helper cells (Figure 5d and Supplementary Figures 9g and h).^22^

**Figure 6:**
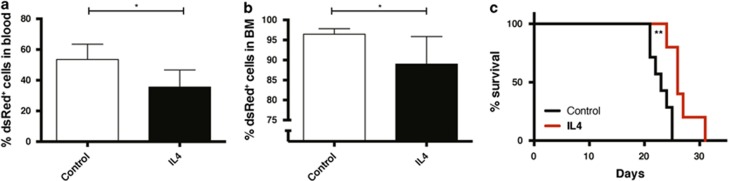
IL4 injections into mice suppresses leukemia burden and increases survival. Daily intraperitoneal injections of mIL4 (60 μg/kg per day) or PBS (control) for 10 days were started 1 day after transplantation of 10 000 c-Kit^+^ *MLL-AF9* leukemia cells into sublethally irradiated mice (six mice per group). (**a**) Percentage of leukemia (dsRed^+^) cells in peripheral blood on day 12 after transplantation. (**b**) Percentage of leukemia (dsRed^+^) cells in bone marrow (BM) on day 13 after transplantation. (**c**) Kaplan–Meier curves showing overall survival of the mice. *P<0.05, **P<0.01.

We next explored whether intraperitoneal injections of IL4 into mice engrafted with AML cells would have antileukemic activity. IL4 injections into mice engrafted with AML cells resulted in lower leukemia burden in both blood (Figure 6a) and bone marrow (Figure 6b), accompanied by a significantly prolonged survival (median 26 versus 23 days, *P*<0.01; Figure 6c). In contrast, upon IL4 injections into healthy mice, no lineage-skewing effects on hematopoietic cells were noted (Supplementary Figure 10). In summary, these findings demonstrate that intraperitoneal injections of IL4 also have a selective antileukemic effect on murine *MLL-AF9* AML cells.

### IL4 induces apoptosis of human AML cells

**Figure 7:**
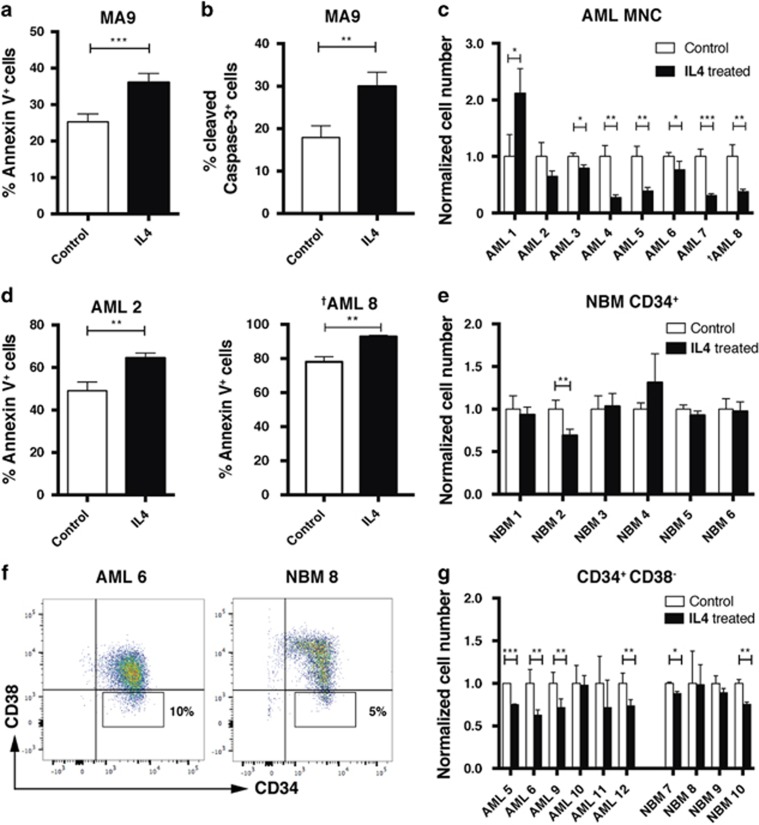
IL4 inhibits human AML cells by inducing apoptosis. Human leukemia cells were cultured for 3 days with hIL4 or without hIL4 (control). (**a–b**) MA9 leukemia cells treated with hIL4. (**a**) Annexin V and (**b**) Caspase-3 staining of MA9 cells (*n*=3). (**c–e**) Primary AML mononuclear cells (MNCs) from patients and NBM CD34^+^ cells were treated with hIL4 (*n*=3). (**c**) Output cell number of seeded AML MNCs following IL4 treatment. Output cell number is normalized to the control within samples. (**d**) Apoptosis analysis of MNCs from two AML samples (AML 2 and 8). (**e**) Output cell number of seeded NBM CD34^+^ cells following IL4 treatment. Output cell number is normalized to the control within samples. (**f**) FACS plots showing CD34 and CD38 staining of representative human CD34^+^ AML and NBM samples. Sorting of CD34^+^CD38^−^ cells was performed according to the depicted gates. (**g**) Output cell number of seeded CD34^+^CD38^−^ AML and NBM cells following treatment with hIL4. Output cell number is normalized to the control within samples. *P<0.05, **P<0.01, ***P<0.001. ^†^Patient with an *MLL-AF9* fusion gene.

To assess whether IL4 has antileukemic activity also in human AML cells, we first stimulated the cytokine-dependent MA9 cell line^23^ with IL4. Similar to the effects we observed on murine *MLL-AF9* AML cells, IL4 stimulation of MA9 cells resulted in suppression of cell proliferation (Supplementary Figure 11a), increased apoptosis via Caspase-3 activation and STAT6 phosphorylation (Figures 7a and b and Supplementary Figure 11b). Moreover, IL4 inhibited the colony formation of the MA9 cells (Supplementary Figure 11c). We next assessed which components of the IL4R complexes that are expressed in AML patient cells using RNA-sequencing data from TCGA.^24^ By contrast to the murine leukemia cells that expressed *Il4ra* and *Il2rg* only, we found that all three receptors are expressed in AML patient cells (Supplementary Figure 12).

We then evaluated whether IL4 has selective antileukemic activity also in primary AML patient cells relative to NBM cells. A majority of the patient samples tested had a normal karyotype, one had an *MLL* rearrangement and several harbored genetic alterations in *FLT3* or *NPM1* (Supplementary Table 2). In short-term cultures (3 days), IL4 treatment resulted in a significant decrease in cell number in six out of eight AML patient samples evaluated (Figure 7c). Similar to the effect observed in the murine AML model and the MA9 cell line, IL4 treatment induced apoptosis of primary AML patient cells (Figure 7d). By contrast, only one out of six NBM CD34^+^ samples responded to IL4 treatment (Figure 7e). Finally, we assessed the effect of IL4 on CD34^+^CD38^−^ AML and NBM cells (Figure 7f), enriched for LSCs and normal hematopoietic stem cells, respectively.^25, 26^ We found that IL4 inhibited the growth and survival of AML CD34^+^CD38^−^ cells in four out of six samples, whereas a milder effect of IL4 was detected on corresponding NBM samples (Figure 7g). These findings show that IL4 induces apoptosis and inhibits cell proliferation in a large proportion of AML patient samples tested, whereas human NBM cells were less affected.

## Discussion

In a leukemic bone marrow, the deregulation of cytokines contributes to an alteration in bone marrow niche architecture, which contributes to leukemic progression, and suppression of normal hematopoiesis.^3, 27, 28, 29, 30^ We here established a cytokine screen using murine fluorescently labeled *MLL-AF9* AML cells and identified IL4 as a selective negative regulator of primitive AML cells. IL4 inhibited the growth and survival of *MLL-AF9* AML cells both in cell cultures and *in vivo* without affecting normal HSPCs. IL4 is mainly known as an anti-inflammatory cytokine that binds to monocytes and suppresses the secretion of proinflammatory cytokines, such as IL1 and tumor necrosis factor-α,^31, 32, 33^ but it also has proinflammatory functions.^34^ Although IL4 has been described to have both positive and negative effects on cancer cells,^35^ the mechanistic basis for the IL4-induced negative effects on cancer cells remains obscure.^36, 37, 38, 39^ Our finding that IL4 induces Caspase-3-mediated apoptosis of AML cells is in agreement with a previously described role for IL4 in inducing apoptosis of monocytes.^40^ However, the finding that IL4 did not alter the cell cycle status of AML cells differs with observations in CLL cells, in which IL4 blocks cell cycle progression into the G1 stage.^41^

Consistent with Stat6 being a downstream mediator of IL4 signaling,^21^ we found that the antileukemic effect of IL4 in AML cells was, at least partially, Stat6-dependent. A similar link between IL4-induced apoptosis and Stat6 has previously been reported in breast cancer, but not in AML.^42^

*Ex vivo* IL4 treatment of leukemia cells suppressed the leukemia-initiating capacities of the cells, partially by inhibiting homing to the bone marrow, suggesting a negative effect of IL4 on LSCs. By contrast, the addition of IL4 to *ex vivo* cultures of normal HSPCs followed by competitive transplantations into mice did not reveal any effect on short- or long-term bone marrow repopulation, nor did it alter the lineage fate of the cells. These findings are in accordance with *Stat6*-deficient hematopoietic stem cells, which are functionally intact, even when challenged in competitive bone marrow repopulation assays.^43^

Importantly, IL4 inhibited AML cells also *in vivo*, with the most prominent antileukemic effect obtained upon retroviral secretion of IL4 by leukemic cells, as compared with intraperitoneal injections of IL4. Most likely, the stronger antileukemic effect obtained upon ectopic secretion of IL4 *in vivo* was attributed to a higher local concentration of IL4 using this strategy. However, because mice in the IL4 group at the time of killing did not have a fully developed leukemia, our data indicate poor tolerability of IL4 overexpression *in vivo*, potentially related to an expansion of T helper cells leading to aberrant cytokine secretion and splenomegaly. The expansion of CD4^+^ T cells was not observed upon intraperitoneal injections of IL4, suggesting that the antileukemic effects of IL4 can be achieved within tolerable doses.

Because IL4 inhibited the growth and survival of human colony-forming MA9 cells and the majority of AML patient samples tested, across several AML genetic and French–American–British subtypes, our data show that the antileukemic effect of IL4 first identified in the mouse model can be translated to human AML. However, to enhance the therapeutic efficacy of IL4, identifying therapies that potentially act in synergy with IL4 are warranted.

In summary, we here demonstrate that IL4 is a negative regulator of primitive AML cells, one of the first cytokines to be identified in this role, and provide *in vitro* and *in vivo* evidence that IL4 has antileukemic activity in AML. We show that IL4 acts by inducing apoptosis of AML cells, and its antileukemic effect is dependent on Stat6. These results highlight IL4 as a suppressor of primitive AML cells, findings that may translate into new therapeutic opportunities in AML.
