# Supplementary material for: Interleukin 4 induces apoptosis of acute myeloid leukemia cells in a Stat6-dependent manner
Source: Leukemia. 2017 Sep 22;32(3):588–96. doi: 10.1038/leu.2017.261 (PMC5843897; doi:10.1038/leu.2017.261)
Supplement: Supplementary Information [file leu2017261x1.docx]

**Supplementary information for:**

**Interleukin 4 induces apoptosis of acute myeloid leukemia cells in a Stat6 dependent manner**

Pablo Peña-Martínez^1^, Mia Eriksson^1^, Ramprasad Ramakrishnan^1^, Marion Chapellier^1^, Carl Högberg^1^, Christina Orsmark-Pietras^1^, Johan Richter^2,3^, Anna Andersson^1^, Thoas Fioretos^1^, and Marcus Järås^1^

^1^Department of Clinical Genetics, Lund University, 22184 Lund, Sweden;

^2^Department of Molecular Medicine and Gene Therapy, Lund University, 22184 Lund, Sweden.

^3^Department of Hematology, Oncology and Radiation Physics, Skåne University Hospital, 22185 Lund, Sweden.

**Supplementary materials and methods**

*Murine leukemia model*

All animal experiments were conducted according to an Animal Care and Use Committee-approved protocol by the Lund/Malmö Ethical Committee. *MLL-AF9* leukemias were generated in a *dsRed* C57BL/6 transgenic background (6051; Jackson Laboratory, Bar Harbor, ME, USA), and in a *Tp53^-/-^* C57BL/6 background (002101; The Jackson Laboratory), as previously described.^1,2^ The *MLL-AF9* leukemias were serially propagated in sublethally irradiated (600 cGy) recipient mice. All experiments involving murine leukemia cells were performed using tertiary or quaternary transplanted leukemia cells. In order to enrich for primitive leukemia cells, femurs from leukemic mice were crushed, red blood cells were lysed using NH_4_Cl solution (StemCell technologies, Vancouver, Canada), and c-Kit^+^ cells were enriched by CD117 MicroBeads in MACS^®^ Cell Separation Columns according to manufacturer’s instructions (Miltenyi Biotec, Bergisch Gladbach, Germany). Except for the propagation of leukemia cells, all experiments involving murine leukemia cells were initiated using c-Kit^+^ bone marrow cells.

*Leukemia cell cultures*

Freshly isolated c-Kit^+^ leukemia cells were plated in serum-free expansion medium (SFEM; Stemspan, StemCell Technologies) supplemented with 1% penicillin/streptomycin. For all experiments involving murine interleukin 3 (mIL3), a concentration of 20 ng/mL was used. The mononuclear cells from primary human samples were cultured in SFEM containing 1% penicillin/streptomycin supplemented with human SCF (hSCF; 20 ng/mL), human thrombopoietin (hTPO;10 ng/mL), and human FLT3 ligand (hFLT3-L; 20 ng/mL). Sorted CD34^+^CD38^-^ NBM and AML cells from patient samples were cultured in Iscove’s modified Dulbecco’s medium (IMDM) supplemented with 1% penicillin/streptomycin, 1% L-glutamine, 0.1 mM β-mercaptoethanol, 15% bovine serum albumin, insulin, transferrin (BIT 9500; StemCell Technologies), hSCF (100 ng/mL), hFLT3-L (50 ng/mL), human IL3 (hIL3; 20 ng/mL), human gCSF (20 ng/mL), StemRegenin-1 (500nM; StemCell Technologies) and UM729 (500 nM; StemCell Technologies). MA9 cells were cultured in IMDM supplemented with 1% penicillin/streptomycin, 1% L-glutamine, 0.1 mM β-mercaptoethanol, 15% fetal bovine serum (FBS), hFLT3-L (20 ng/mL), hTPO (10 ng/mL), hSCF (20 ng/mL), hIL3 (10 ng/mL) and human IL6 (hIL6; 20 ng/mL). Except for the IL4-dose-titration experiments, murine or human IL4 was added to the culture medium at a concentration of 100 ng/mL.

*Colony forming assay*

1 000 c-Kit^+^ dsRed^+^ murine *MLL-AF9* leukemia cells were plated in triplicates in 1 mL of Methocult™ GF M3434 medium (StemCell Technologies) with or without mIL4 (100 ng/mL). 1 000 human MA9 cells were cultured in Methocult™ H4435 enriched medium (StemCell Technologies), supplemented with 100 ng/mL of hIL4 or without hIL4 (control). Plates were incubated at 37° C and colony number was determined after 10 days.

*Homing assay*

c-Kit^+^ dsRed^+^ leukemia cells were cultured for 3 days *ex vivo* with mIL4 and mIL3 or mIL3 as control, then the cells were counted using CountBright beads (Life Technologies, Carlsbad, CA, USA) and stained with APC–Annexin V (BioLegend, San Diego, CA, USA) as described in the apoptosis analysis. Sublethally irradiated mice were transplanted with 3 000 000 or 5 000 000 viable (Annexin V^-^) cells. The frequency of leukemia (dsRed^+^) cells was determined by flow cytometric analysis of bone marrow cells harvested from femurs 24 hours post transplantation.

*Cell cycle analysis*

To determine the cell cycle status of cells treated with IL4, the cells were harvested after 72 hours of culture, then fixed in 1.6% paraformaldehyde for 10 minutes, and permeabilized after washing with 1 mL of chilled ethanol. Subsequently, the cells were washed twice in phosphate-buffered saline (PBS) containing 2% FBS and stained with a Ki67–APC antibody (Miltenyi Biotec), and DAPI (BioLegend) before analysis by flow cytometry.

*Apoptosis and Caspase-3 analysis*

Leukemia cells cultured for 72 hours were harvested, washed in 1 x binding buffer (BD Biosciences), and stained with APC–Annexin V (BioLegend) and 7-AAD (BD Biosciences) or DAPI (BioLegend) for 20 minutes at room temperature. The percentages of Annexin V^+^ and 7-AAD^+^/DAPI^+^ cells were determined by flow cytometric analysis. For the Caspase-3 analysis, cells were fixed and permeabilized as described for the cell cycle analysis, and stained for 30 minutes at 4°C using a PE–conjugated antibody (Cell Signaling Technology, Danvers, MA, USA) detecting cleaved (active) Caspase-3.

*Competitive bone marrow transplantations*

For donor cells, bone marrow cells from B6SJL (CD45.1^+^) and C57BL/6 x B6SJL (CD45.1^+^CD45.2^+^) mice were harvested and c-Kit^+^ cells selected using magnetic beads as described above for the leukemia cells. Lin^–^Sca1^+^c-Kit^+^ (LSK) cells were sorted and cultured *ex vivo* for 3 days in SFEM culture medium supplemented with murine SCF (mSCF; 20 ng/mL) and murine TPO (mTPO; 50 ng/mL). The CD45.1^+^CD45.2^+^ LSK cells also received mIL4 (100 ng/mL). After culture, cells corresponding to 10 000 seeded cells from each condition were mixed and transplanted into C57BL/6 (CD45.2^+^) mice, which had been lethally irradiated (900 cGy), together with 200 000 CD45.2^+^ bone marrow support cells. Blood samples were taken at weeks 4, 8, and 16 after transplantation. No blinding or randomization was performed, but all mice were age and gender matched.

# Flow cytometric analysis and cell sorting

Prior to sorting the LSK cells, c-Kit^+^ bone marrow cells were stained with APC–c-Kit, Pacific Blue–Sca1, and PE/Cy5–Lin^–^ cocktail antibodies (all from BioLegend). In the competitive bone marrow transplantation experiments and following mIL4 injections into healthy mice, lineage stains were performed using the following antibodies: PE/Cy5–Ter119, PE–CD3, APC/Cy7–Gr1, BV510–CD11b, PE/Cy7–B220, APC–CD45.1, BV421–CD45.2, and propidium iodide was used as a viability marker (all from BioLegend). To analyze B and T cell populations in mice transplanted with leukemia cells transduced with the MIG–IL4 retroviral vector, bone marrow and spleen cells were stained using APC–CD3, PECy7–CD4, BV510–CD8 and PerCP/Cy5.5–B220 antibodies (all from BioLegend). Sorting of CD34^+^CD38^-^ AML and NBM cells was performed following staining with CD34–APC/Cy7 and CD38–BV450 antibodies (all from Biolegend). Sorting of IL4ra^high^ and IL4ra^low^ was performed following staining with an IL4ra–BV421 antibody (BD biosciences). For detection of Stat6 and phosphorylated Stat6 (pStat6), leukemia cells were cultured in SFEM without cytokines for 2 hours, then mIL4 was added to the culture medium and cells were collected after 2 hours. The cells were fixed and permeabilized as described for the cell cycle analysis and stained with AF647–pStat6 (pY641) or AF647–Stat6 antibodies (BD Biosciences) on ice for 1 hour before analysis by flow cytometry.

# Viral vector generation and production

The murine stem cell virus gammaretroviral vector coexpressing a m*IL4* cDNA (GenScript, Piscataway, NJ, USA; gene synthesis) and *GFP* connected with an internal ribosome entry site (MIG–IL4) was generated by ligation using *Xho*1/*Eco*R1 restriction sites. The *Cas9*-expressing lentiviral vector coexpressing a puromycin resistance gene was obtained from Addgene (#58329; Cambridge, MA, USA). A lentiviral vector expressing a single-guide RNA (sgRNA) directed to *Stat6* was generated by ordering oligonucleotides complementary to *Stat6* (Life Technologies). The sgRNA sequence was designed using the online tool CRISPR Design (Broad Institute, http://crispr.mit.edu/) targeting exon 3 (*Stat6* sgRNA: AGAAAGCATCTGAACCGACC), and shuttled into the pLKO5.sgRNA.EFS.GFP vector (Addgene, #57822). The control sgRNA (GTCTTCGGAGGACAGTACTC) was designed to not target any endogenous gene. Viral vectors were produced using standard protocols in 293T cells. Gammaretroviral vectors were pseudotyped with an ecotropic envelope and lentiviral vectors with a vesicular stomatitis virus G envelope. For transduction experiments, the medium was supplemented with mIL3 (20 ng/mL), mSCF (50 ng/mL), and hIL6 (20 ng/mL) and mixed with the viral vectors. Transduction was performed by spinoculation at 600 x g for 1 hour at 32°C.

*CRISPR/Cas9*

Cas9^+^ leukemia cells were generated by transducing the *MLL-AF9* murine leukemia cells with the *Cas9*-expressing lentiviral vector. To expand the Cas9^+^ *MLL-AF9* leukemia cells, transduced cells were treated with puromycin at a concentration of 1 μg/mL for 2 days before transplantation into sublethally irradiated mice. When mice succumbed to disease, the Cas9^+^ *MLL-AF9* leukemia cells were harvested and enriched for primitive cells using c-Kit selection. Subsequently, the *Stat6* sgRNA and a control sgRNA lacking an endogenous target gene were expressed by lentiviral vectors in the Cas9^+^ *MLL-AF9* leukemia cells. The genetic changes introduced by the CRISPR/Cas9 system into the *Stat6* gene were quantified by next-generation sequencing using the online tool CRISPResso (Harvard School of Public Health, http://crispresso.rocks/).^3^

*Animal experiments*

The IL4 dose administered to the mice (60 µg/kg per day) via intraperitoneal injections was transformed according to the Food and Drug Administration (FDA) guidelines from a human phase II trial in which IL4 was given to patients with B-cell neoplasms.^4,5^ All mice in the survival experiments were sacrificed based on at least one of the following criteria: immobility, hunched back, hind leg paralysis, or dehydration. No blinding or randomization was performed, but all mice were age and gender matched.

# ELISA

The concentration of mIL4 was determined using enzyme-linked immunosorbent assay (ELISA) kits (Thermo Scientific, Waltham, MA, USA) according to the manufacturer’s instructions. Serum samples for the ELISA analysis were prepared by centrifuging blood samples at 10 000 x g and collecting the supernatant. Bone marrow supernatants were prepared by flushing the femurs with 100 μl PBS. An Infinite F50 absorbance reader (Tecan, Männedorf, Switzerland) instrument was used for the analysis.

# RNA sequencing analysis

RNA was extracted using the QIAshredder and RNeasy Microkit (QIAgen, Hilden, Germany), and the RNA quality was validated in the 2100 Bioanalyzer (Agilent Technologies, Inc., Santa Clara, CA, USA). RNA libraries from mouse cells were prepared using the TruSeq RNA sample prep kit v2 (Illumina, San Diego, CA, USA) and sequenced in a NextSeq 500 Desktop Sequencer (Illumina) using the NextSeq 500/550 Mid Output v2 kit, 150 cycles (Illumina). The sequenced reads were aligned to the mm10 reference mouse genome using TopHat 2.0.13.^6^ Statistical analysis, differential gene expression, and visualization of the RNA sequencing data were performed using Qlucore Omics Explorer 3.0 (Qlucore, Lund, Sweden). Lists of differentially expressed genes within AML and NBM samples were generated separately by a two group comparison between IL4-treated and control cells using an FDR of 0.05 as significance cut-off. Gene set enrichment analysis (GSEA)^7^ was performed with pre-ranked gene lists, based on the GSEA guidelines for RNA sequencing data, following *t*-test comparison between gene expression levels in IL4-treated versus non-treated cells (control).

## Figure S1


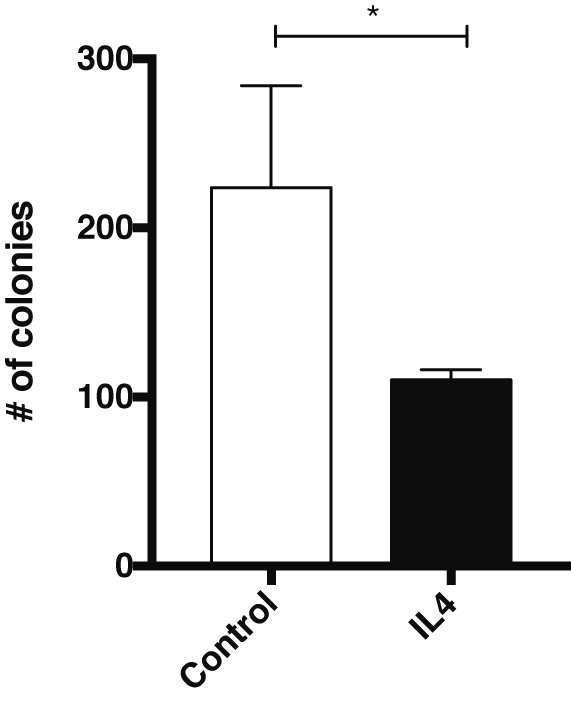


**Figure S1. IL4 inhibits the colony forming potential of AML cells.** 1 000 *MLL-AF9* leukemia cells were plated in triplicates and mIL4 (100 ng/mL) was added to the methocellulose medium. Colony number was quantified after 10 days (n=3). **P*<.05

## Figure S2


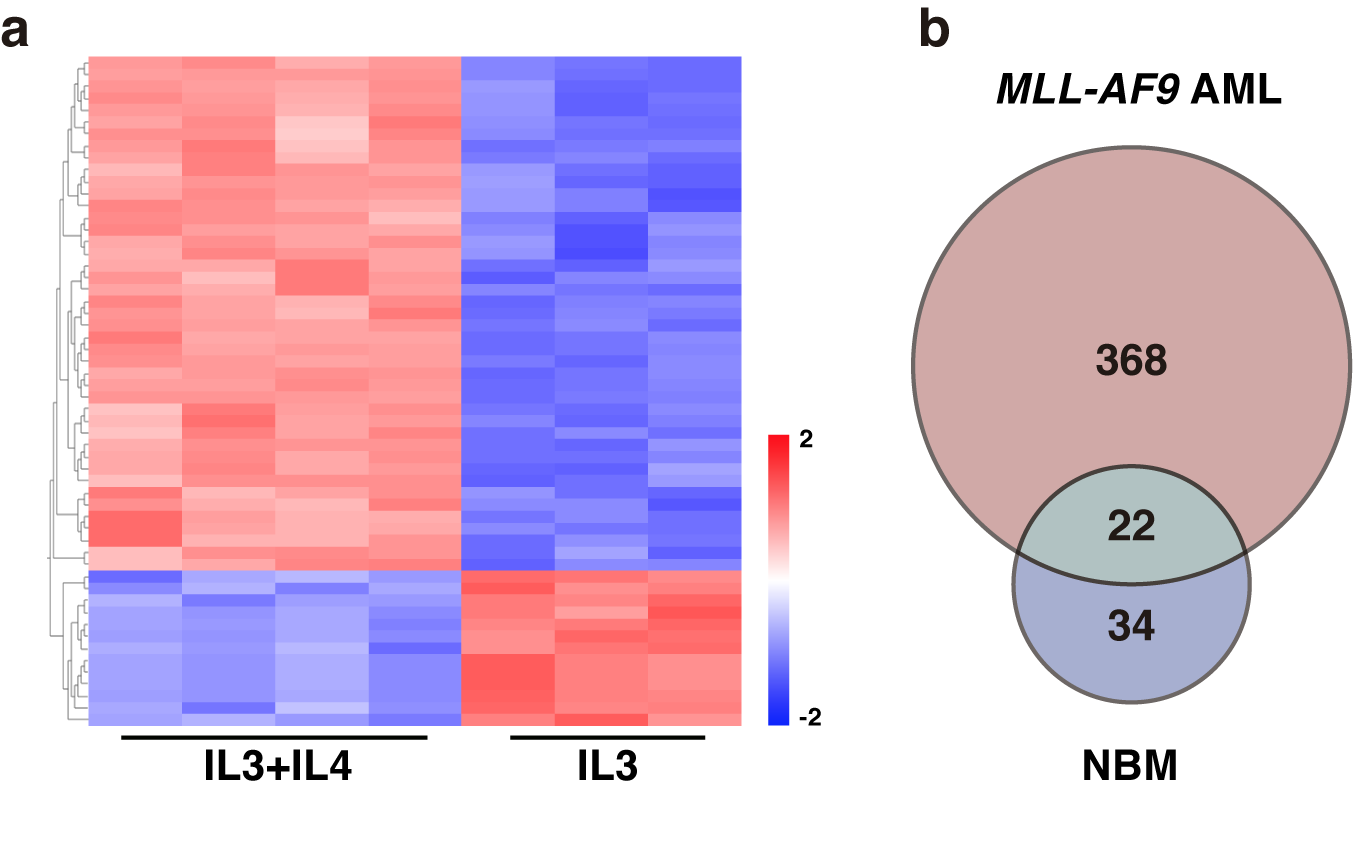


**Figure S2. IL4 induces distinct gene expression patterns in NBM and AML cells.** RNA sequencing was performed on murine c-Kit^+^ NBM cells stimulated for 18 hours with IL4 and IL3 or IL3 only (control). **(a)** A two-group comparison between control and IL4-treated NBM cells revealed an IL4-induced signature of 56 differentially expressed genes (FDR<0.05, *P*<0.001). Genes in the heatmap are ordered by hierarchical clustering. **(b)** Overlap between the differentially expressed genes in AML and NBM cells upon IL4 treatment. Only 22 genes (5.6% of all genes in the AML group) were shared between the two populations.

## Figure S3

**
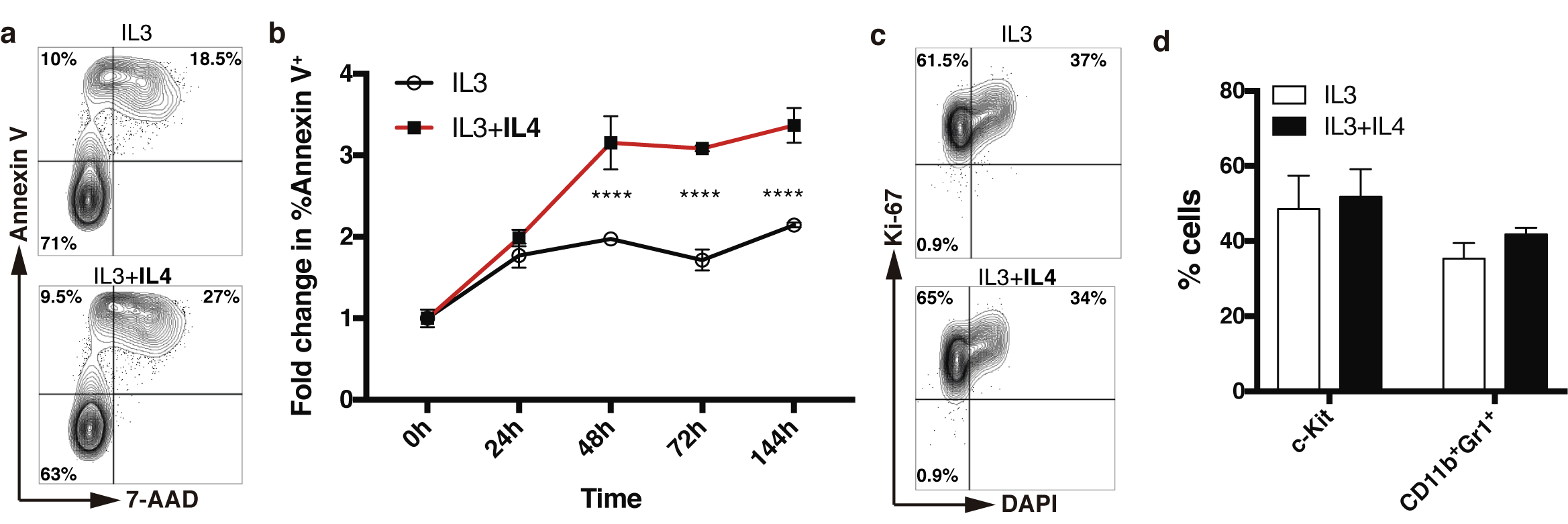
**

**Figure S3. Apoptosis, cell cycle, and differentiation analyses of *MLL-AF9* leukemia cells treated with IL4.** *MLL-AF9* murine leukemia cells were treated with mIL4 and mIL3 or mIL3 only (control) for three days before apoptosis, cell cycle and differentiation analyses were perfomed (n=3). **(a)** Representative FACS contour plots of the apoptosis analysis showing Annexin V and 7-AAD staining. **(b)** Fold change in the percentages of Annexin V^+^ cells during mIL4 treatment for 6 days relative to day 0 (n=3). **(c)** Representative FACS contour plots of the cell cycle analysis showing Ki-67 and DAPI staining of leukemia cells. Upper left quadrant includes cells in the G1 phase, upper right in the S/G2/M phase, and lower left in the G0 phase. **(d)** Differentiation analysis of cells after treatment with mIL4 for three days. mIL4 was used at 100 ng/mL and mIL3 at 20 ng/mL. *****P*<.0001.

## Figure S4


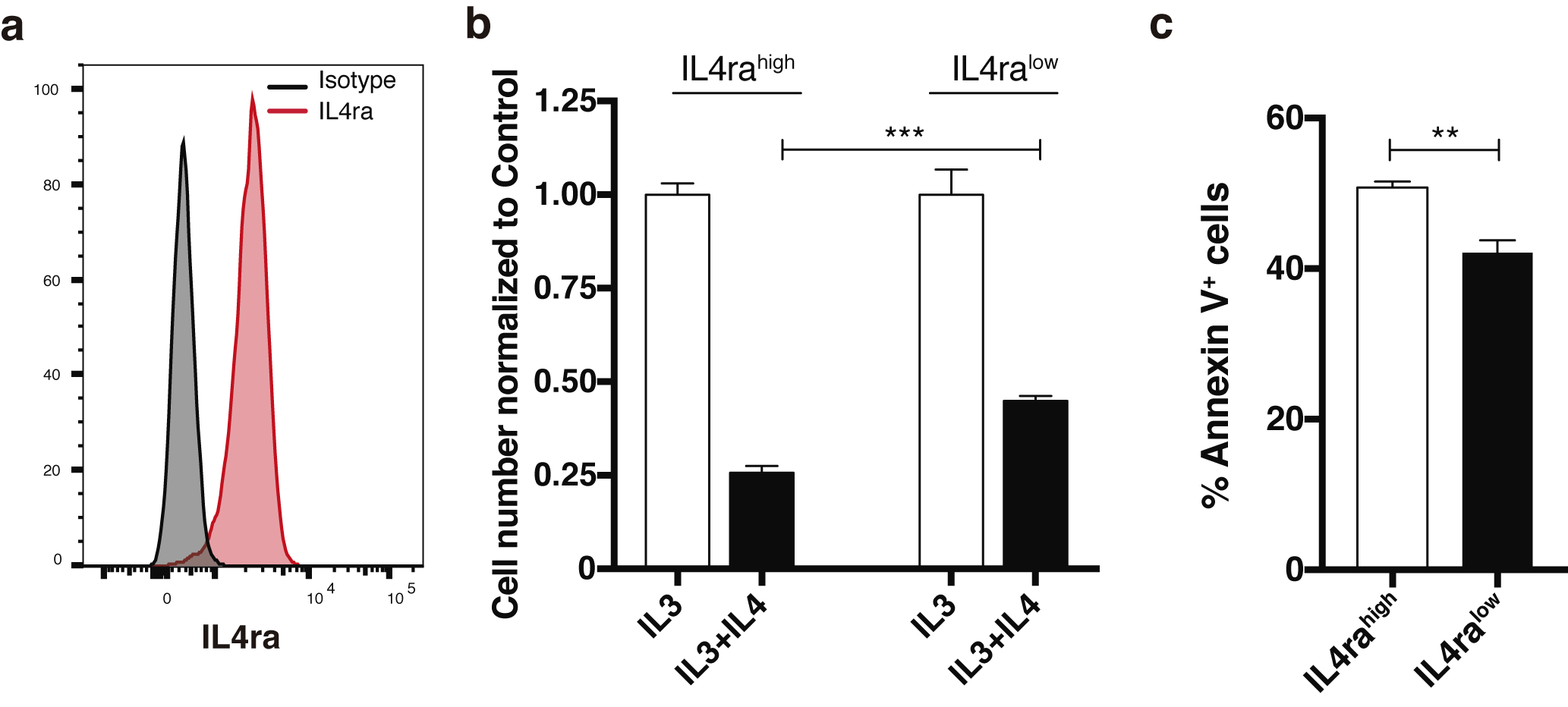


**Figure S4. IL4ra expression levels sensitizes leukemia cells to IL4.** **(a)** Staining of IL4ra in dsRed^+^ *MLL-AF9* leukemia cells using flow cytometry. **(b-c)** Leukemia cells were sorted according to IL4ra expression (IL4ra^low^: 10% lowest IL4ra expression; IL4ra^high^: 10% highest IL4ra expression) and treated for 3 days with or without mIL4 (control). **(b)** Cell number normalized to the control for each population and **(c)** percent Annexin V^+^ cells of the IL4 treated groups (n=3). ***P*<.01, ****P*<.001.

## Figure S5

**Figure S5. Expression of IL4 receptor subunits in murine *MLL-AF9* leukemia cells.** RNA sequencing was performed on freshly isolated murine c-Kit^+^ AML cells, and the read counts were normalized using the trimmed mean of M-values (TMM) method (n=4). Data presented as box and whiskers diagrams, the line indicates median, box limits are first and third quartiles and bars indicate maximum and minimum values. n.d.: not detected.

## Figure S6

**Figure S6. *Ex vivo* IL4 treatment inhibits leukemia-initiating cells.** *MLL-AF9* murine c-Kit^+^ leukemia cells were treated with mIL4 and mIL3 or mIL3 only (control) for 3 days, then 10 000 viable leukemia cells were transplantated into sublethally irradiated recipient mice for both groups (10 mice per group). Kaplan–Meier curves show the survival of the mice.

## Figure S7


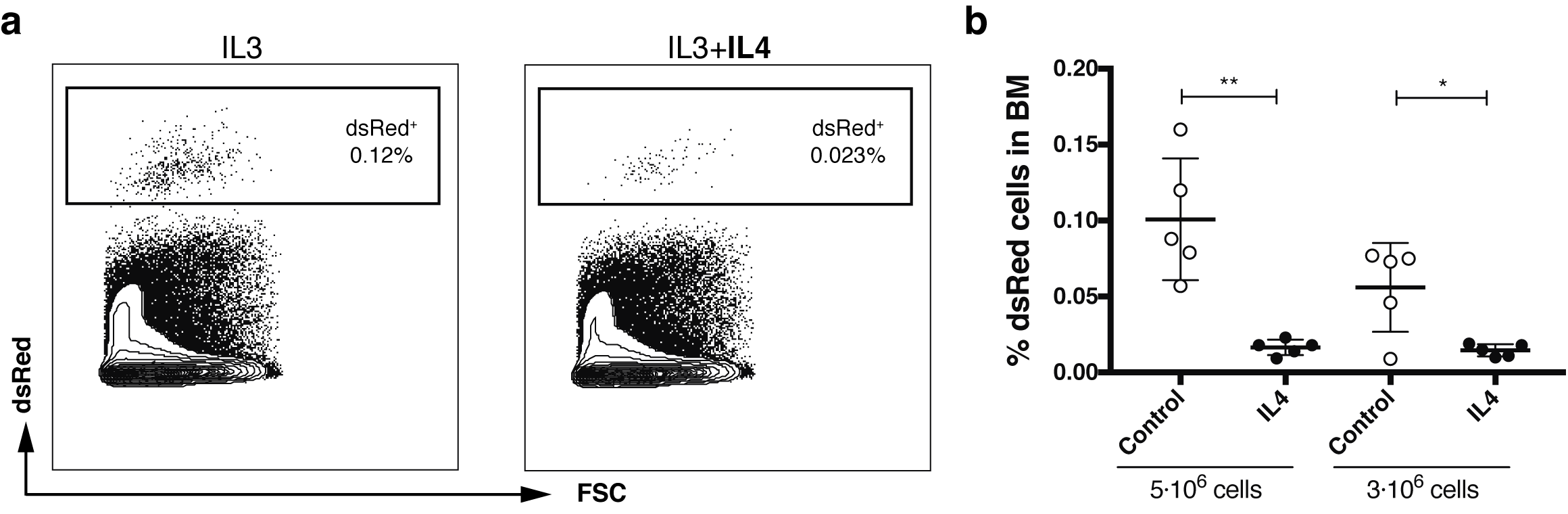


**Figure S7. *Ex vivo* treatment with IL4 inhibits bone marrow homing of AML cells.** *MLL-AF9* leukemia cells were treated with mIL4 and mIL3 or mIL3 only (control) for 3 days before transplantation into recipient mice (5 mice per group). The number of cells transplanted per mouse is indicated in the figure. **(a)** Representative FACS contour plots showing the percentage of dsRed^+^ *MLL-AF9* leukemia cells in the bone marrow 24 hours post transplantation. **(b)** Percentage of leukemia cells in the bone marrow 24 hours post transplantation. **P*<.05, ***P*<.01.

## Figure S8


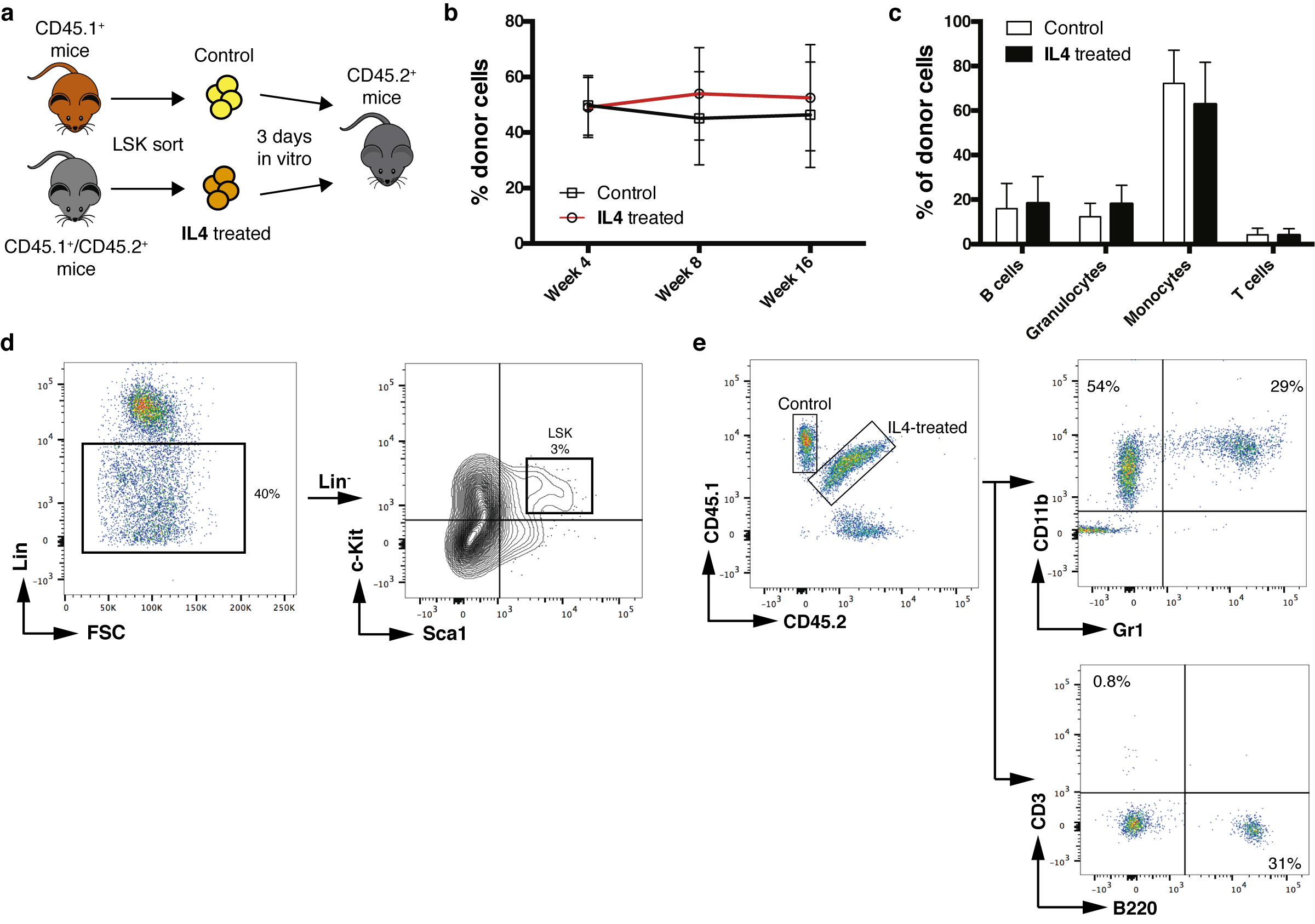


**Figure S8. *Ex vivo* IL4 treatment did not affect normal HPSCs or their lineage output as assessed in competitive bone marrow transplantation assays** **(a)** Schematic diagram showing the experimental design of the competitive bone marrow repopulation assay. LSK cells from CD45.1^+^ mice were cultured without mIL4 (control) and LSK cells from CD45.1^+^/CD45.2^+^ cells were cultured with mIL4. After 3 days of *ex vivo* culture, cells from the two conditions were mixed and transplanted into lethally irradiated mice together with bone marrow support cells (CD45.2^+^) (12 mice per group). mTPO (50 ng/mL) and mSCF (20 ng/mL) were used as the baseline cytokine condition. **(b)** Donor contribution of IL4-treated and control cells over time in recipient mice. **(c)** Percentage of B cells (B220^+^), granulocytes (CD11b^+^Gr-1^+^), monocytes (CD11b^+^Gr-1^-^), and T cells (CD3^+^) at week 16 in peripheral blood within each donor population. mIL4 was used at 100 ng/mL and mIL3 at 20 ng/mL. **(d)** FACS profiles showing gating of LSK bone marrow cells **(e)** Gating of donor cells (Control, CD45.1^+^/CD45.2^-^; IL4 treatment group, CD45.1^+^/CD45.2^+^) from a representative mouse 16 weeks after transplantation (left panel). Percentages of CD11b^+^/Gr-1^+^ (upper right panel) and CD3^+^/B220^+^ (lower right panel) cell populations were determined within each donor population.

## Figure S9


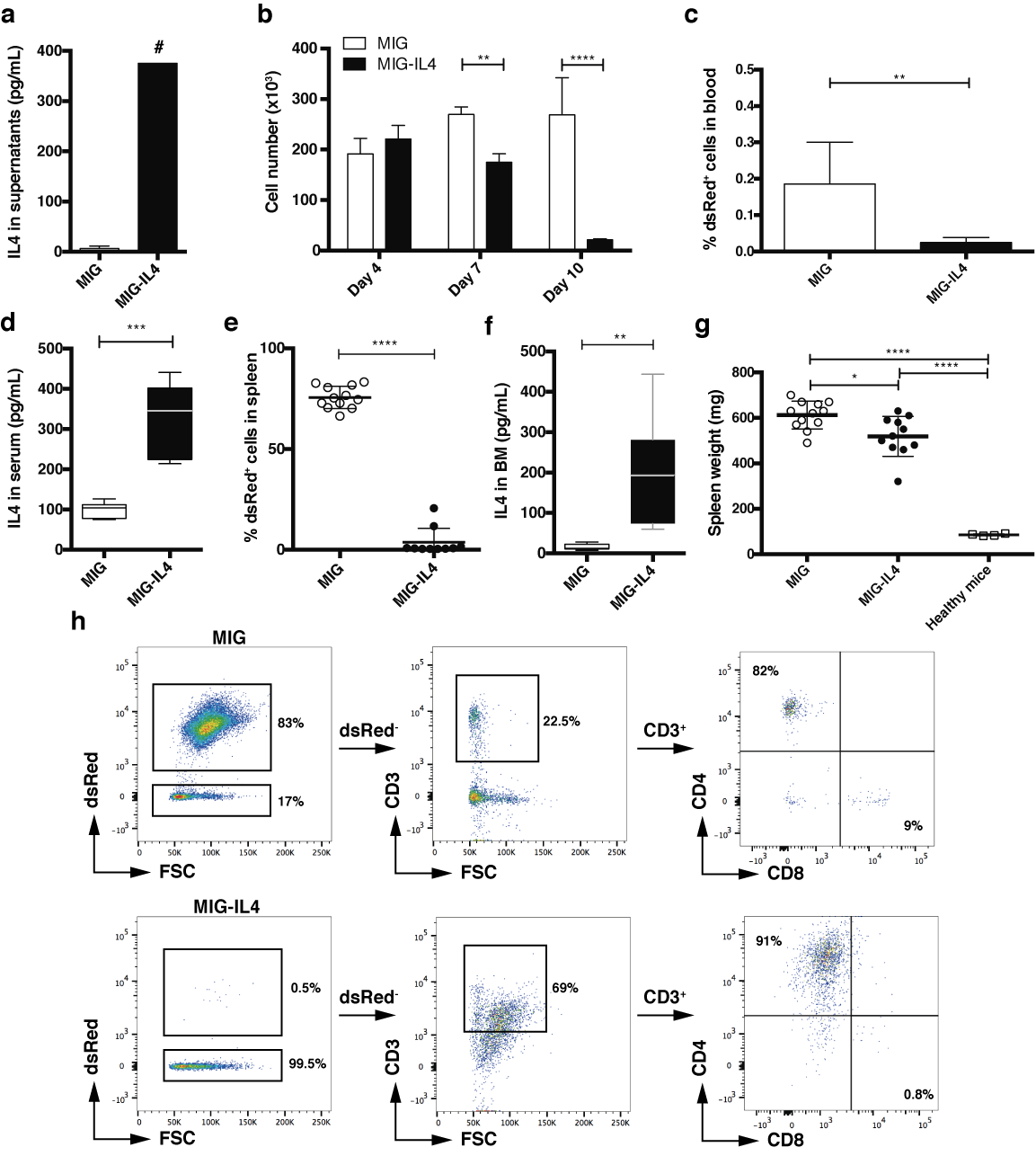


**Figure S9. IL4 overexpression in *MLL-AF9* leukemia cells.** c-Kit^+^ *MLL-AF9* leukemia cells were transduced with retroviral vectors expressing a mIL4 cDNA (MIG–IL4) or a control vector (MIG). **(a)** IL4 levels in the supernatants of leukemia cells cultured for 7 days post transduction. IL4 levels in the MIG–IL4 group were out of range for the ELISA kit used (#>375 pg/mL; n=3). **(b)** Cell number of MIG–IL4 or MIG transduced leukemia cells cultured for ten days with mIL3 (n=3). **(c)** Percentage of dsRed^+^ (leukemia) cells in the peripheral blood of mice 2 weeks after transplantation with sorted GFP^+^ leukemia cells following MIG–IL4 or MIG transduction (6 mice per group). **(d)** IL4 levels in peripheral blood 2 weeks after transplantations with sorted GFP^+^ leukemia cells following MIG–IL4 or MIG transduction. **(e)** Percentage of leukemia (dsRed^+^) cells in the spleens at the time of sacrifice. **(f)** IL4 levels in bone marrow at the moment of sacrifice based on ELISA measurements (6 mice per group). **(g)** Spleen weight of mice transplanted with sorted GFP^+^ leukemia cells transduced with the MIG–IL4 (11 mice) or the MIG vector (12 mice). Four healthy mice that were not transplanted with leukemia cells were included as an additional control. **(h)** Flow cytometric analysis of spleen cells from mice at the time of sacrifice that had been transplanted with GFP^+^ leukemia cells following MIG–IL4 or MIG transduction. Non-leukemic cells (dsRed^-^) cells were gated and the percentages of T helper cells (CD4^+^) and cytotoxic T cells (CD8^+^) were determined within the CD3^+^ population. In the box and whiskers plots, the white line indicates median, box limits are first and third quartiles and bars indicate maximum and minimum values.**P*<.05, ***P*<.01, ****P*<.001, *****P*<.0001.

## Figure S10


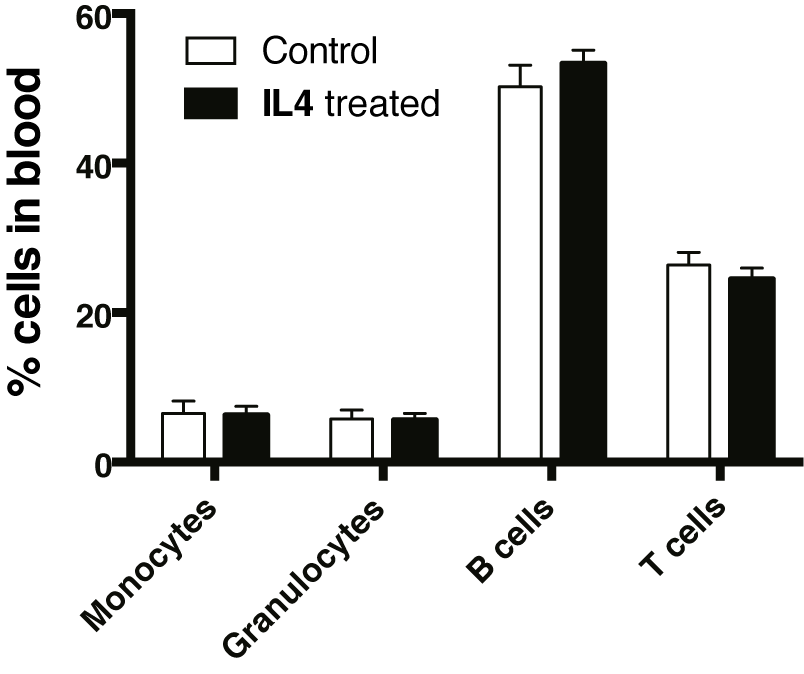


**Figure S10. Distribution of blood lineages in non-leukemic mice treated with IL4.** Percentage of B cells (B220^+^), granulocytes (CD11b^+^Gr-1^+^), monocytes (CD11b^+^Gr-1^-^), and T cells (CD3^+^) in mice treated daily for 10 days with i.p. injections of mIL4 or PBS as control (6 mice per group). The mice had not received leukemia cells.

## Figure S11


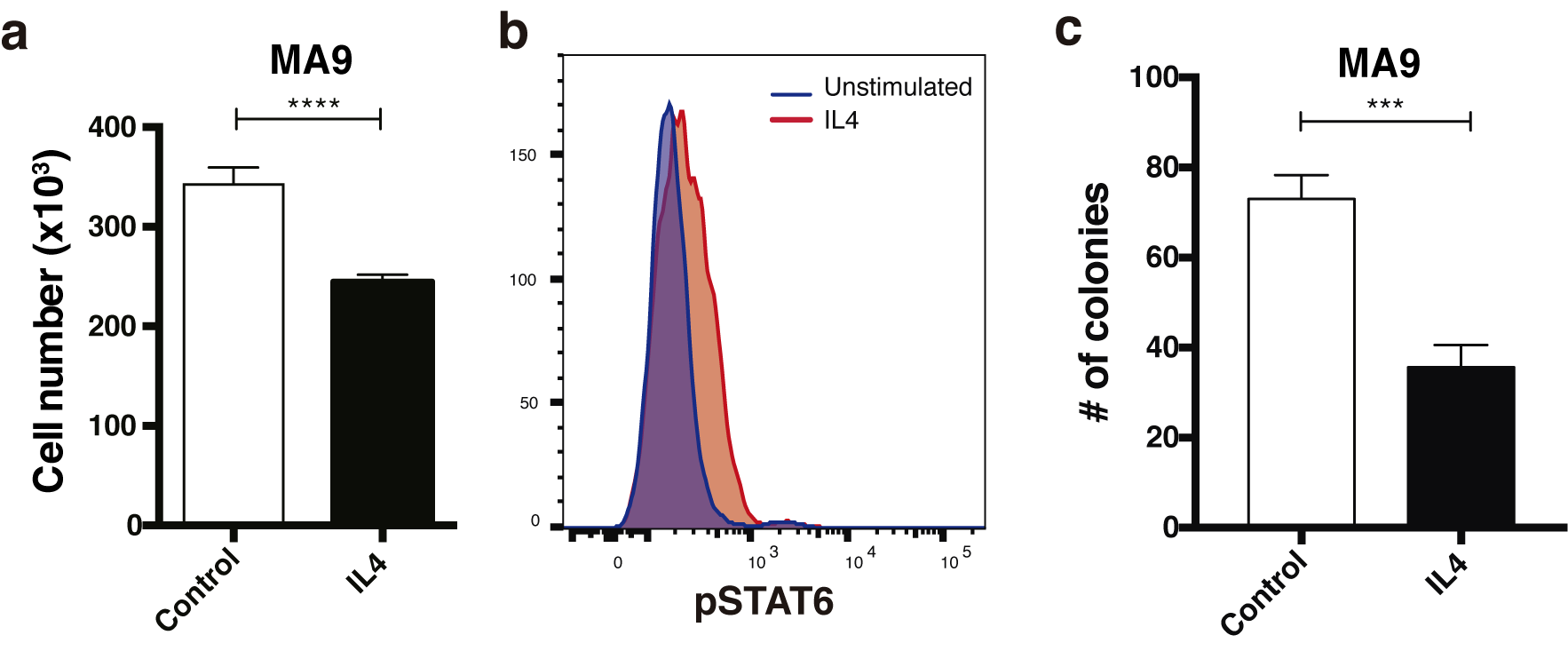


**Figure S11. IL4 induces Stat6 phosphorylation, inhibits cell proliferation and colony formation of MA9. (a)** Human MA9 leukemia cells were treated for 3 days with hIL4 (100 ng/mL) or no treatment (control), then cell number was quantified by FACS using CountBright counting beads (n=3). **(b)** Representative histogram showing pSTAT6-staining in MA9 cells prestimulated with hIL4 for 2 hours. **(c)** Colony forming assay with MA9 cells. 1 000 MA9 cells were seeded with or without hIL4 in methocellulose cultures (n=3). ****P*<.001, *****P*<.0001.

## Figure S12

**Figure S12. Expression of IL4 receptor subunits in AML patient samples**. RPKM (reads per kilobase per million mapped reads) values for the IL4R subunits in human AML samples (n=173) were obtained from RNA sequencing data from TCGA. Data presented as box and whiskers diagrams showing the logarithm of the RPKM values. The line indicates median, box limits are first and third quartiles and bars indicate maximum and minimum values.

##

**Table S1. List of the 114 cytokines included in the screen and screening results.** Presented are the sources of the cytokines, the average cell count and standard deviation (SD; n=3) after 3 days' culture, and the ratio in cell count between *MLL-AF9* leukemia and NBM cells.


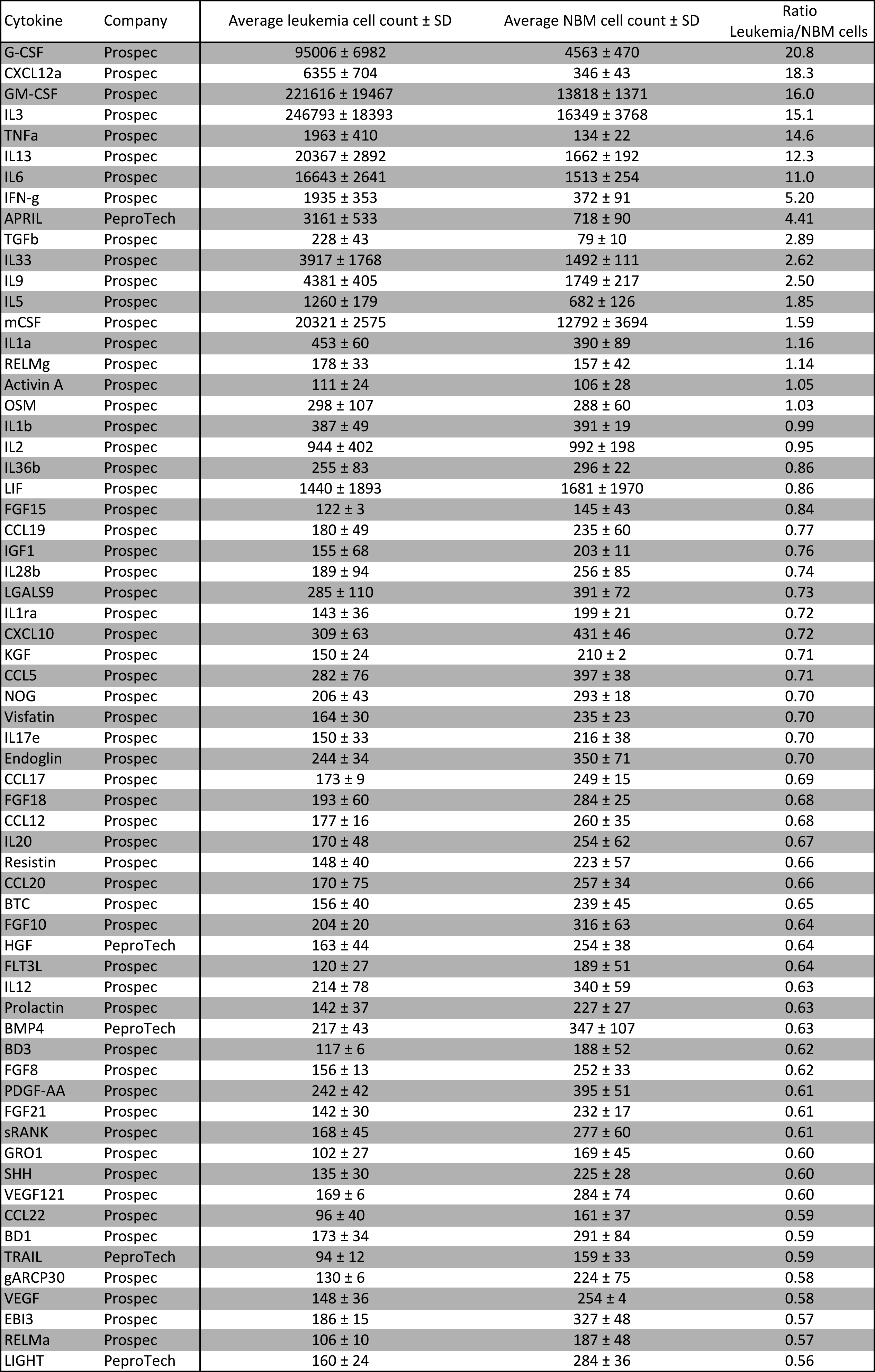


**Table S1,** continued**.**

**Table S2. Patient characteristics.**

**
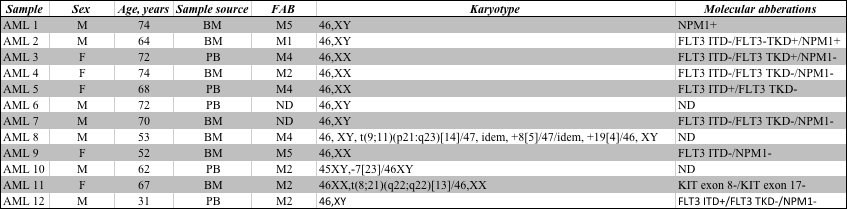
**

Abbreviations: FAB, French–American–British classification; M, male; BM, bone marrow; F, female; PB, peripheral blood; ND, not determined.

**Table S3. Gene sets enriched in the IL4 signature of AML cells (FDR<0.05), and data for the same gene sets matched with the IL4-signature in NBM cells.**

**
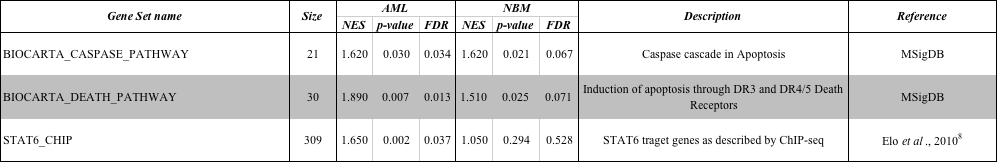
**

Abbreviations: NES, normalized enrichment score; FDR, false-discovery rate; MsigDB, molecular signatures database.

**Table S4. Genes included in the gene sets enriched in the IL4 signature of AML cells.**

**
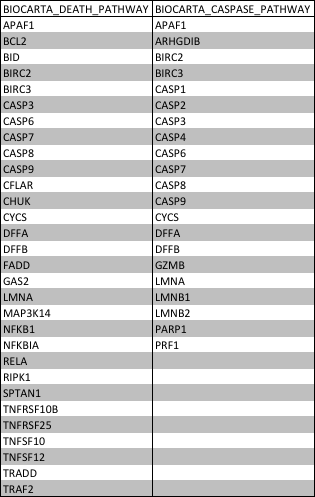
**

**References**

1. Krivtsov AV, Twomey D, Feng Z, Stubbs MC, Wang Y, Faber J*, et al.* Transformation from committed progenitor to leukaemia stem cell initiated by MLL–AF9. *Nature* 2006; **442**(7104)**:** 818-822.

2. Järås M, Miller PG, Chu LP, Puram RV, Fink EC, Schneider RK*, et al.* Csnk1a1 inhibition has p53-dependent therapeutic efficacy in acute myeloid leukemia. *J Exp Med* 2014 Apr 7; **211**(4)**:** 605-612.

3. Pinello L, Canver MC, Hoban MD, Orkin SH, Kohn DB, Bauer DE*, et al.* CRISPResso: sequencing analysis toolbox for CRISPR-Cas9 genome editing. *bioRxiv* 2015.

4. Wiernik PH, Dutcher JP, Yao X, et al. Phase II study of interleukin-4 in indolent B-cell non-Hodgkin lymphoma and B-cell chronic lymphocytic leukemia: a study of the Eastern Cooperative Oncology Group (E5Y92). *J Immunother*. 2010;33(9):1006-1009.

5. FDA. Guidance for industry-Estimating the maximum safe dose in initial clinical trials for therapeutics in adult healthy volunteers.; 2005.

6. Kim D, Pertea G, Trapnell C, Pimentel H, Kelley R, Salzberg SL. TopHat2: accurate alignment of transcriptomes in the presence of insertions, deletions and gene fusions. *Genome Biol*. 2013;14(4):R36.

7. Subramanian A, Tamayo P, Mootha VK, et al. Gene set enrichment analysis: A knowledge-based approach for interpreting genome-wide expression profiles. *Proc Natl Acad Sci U S A*. 2005;102(43):15545-15550.

8. Elo LL, Jarvenpaa H, Tuomela S, et al. Genome-wide profiling of interleukin-4 and STAT6 transcription factor regulation of human Th2 cell programming. *Immunity*. 2010;32(6):852-862.
